# Toddlers Are Happier Giving to Others Than to Themselves

**DOI:** 10.1111/desc.70171

**Published:** 2026-03-17

**Authors:** Enda Tan, Julia Van de Vondervoort, Jeneesha Dhaliwal, Lara B. Aknin, Jane Kiley Hamlin

**Affiliations:** ^1^ Department of Psychology University of British Columbia Vancouver Canada; ^2^ Department of Psychology Simon Fraser University Burnaby Canada

**Keywords:** emotion, happiness, prosocial behavior, sharing, toddlers, warm glow

## Abstract

Humans routinely share valuable resources, even at significant personal cost (e.g., organ donation). What motivates such generosity? This study examined the emotional benefits of sharing in toddlers with the largest and youngest sample to date (*N* = 134; *M_age_* = 20.50 months, range = 16.57–23.77 months). Replicating prior studies, toddlers displayed greater happiness after giving than receiving. Extending these findings, we demonstrate that sharing resources provided by an experimenter elicited greater happiness than observing the experimenter share, suggesting that actively performing prosocial acts leads to greater reward. Crucially, we ruled out alternative explanations by demonstrating that the emotional benefits of sharing are not attributable in this study to emotional contagion (toddlers’ happiness was unrelated to recipient's enthusiasm) or following an experimenter's instructions (toddlers were happier giving to others than to themselves). These findings provide evidence that sharing is intrinsically rewarding from shortly after it first emerges, which may serve as a proximate mechanism driving cooperation across societies.

**Summary:**

- We provide evidence that sharing is intrinsically rewarding soon after this behavior emerges in ontogeny.
- Toddlers displayed greater happiness after giving than receiving, and actively sharing resources led to more happiness than merely observing sharing.
- Giving to others elicited more happiness than giving to oneself, suggesting that sharing is emotionally rewarding due to its prosocial nature.

## 1 Introduction

A defining feature of human society is the widespread practice of generous sharing and other prosocial acts (Axelrod 1984; Fehr and Fischbacher 2003). Indeed, people readily offer valuable resources to others, whether it is giving food to neighbors, providing expertise to colleagues, or donating to charities. These behaviors can involve considerable personal costs, as seen in remarkable acts of generosity such as surrogate pregnancy and organ donation. This widespread willingness to share, even at significant expense to oneself, raises a question: What motivates such behaviors? Here, we present evidence that sharing is emotionally rewarding from shortly after the first birthday (when sharing first emerges and becomes more consistent), and that these emotional benefits are uniquely tied to the prosocial nature of sharing, rather than simply following the direction/request of an adult or authority figure. These emotional rewards may serve as a proximate mechanism for promoting sharing and other prosocial behaviors across societies.

Sharing emerges early in the second year of life and increases in frequency as children age (Brownell et al. 2013; Dunfield et al. 2011; Hay 1979; House et al. 2013; Rheingold et al. 1976; Svetlova et al. 2010). One explanation for this development is that sharing and other prosocial behaviors elicit happiness in the giver, creating a positive feedback loop that reinforces the behavior and prompts future acts of generosity (Aknin, Dunn, et al. 2012, Aknin et al. 2018; Hui 2022; Yang 2024). Supporting this idea, research with adolescents and adults has shown everyday acts of prosociality (e.g., helping, comforting, sharing) are associated with higher self‐reported happiness and well‐being (Cash et al. 2024; Gregori et al. 2024). Additionally, individuals assigned to engage in prosocial (e.g., spending money on others) versus self‐focused activities reported greater positive emotions, suggesting a causal link between prosocial acts and happiness (Aknin, Barrington‐Leigh, et al. 2013, Aknin et al. 2015; Dunn et al. 2008; Martela and Ryan 2016; Nelson et al. 2016; for reviews, see Aknin et al. 2022; Curry et al. 2018; Hui et al. 2020). Similarly, toddlers and preschoolers across cultures showed greater happiness (as rated by independent observers) after following an experimenter's instructions to give resources (e.g., treats, stickers) to others than after receiving resources themselves (Aknin, Hamlin, et al. 2012, Aknin et al. 2015; Fast et al. 2023; Song et al. 2020; see also Wu et al. 2017), suggesting young children find giving more rewarding than receiving. Further, preschoolers were happier after giving than after merely observing others give (Fast et al. 2023). Finally, toddlers were happier after giving resources they were previously told were theirs (e.g., “costly giving”) versus the experimenter's (e.g., “non‐costly giving”; Aknin, Hamlin, et al. 2012; Aknin et al. 2015; Song et al. 2020; note preschoolers did not show this effect), suggesting actively performing an act of giving, and giving that is genuinely prosocial, leads to greater happiness (see also Hepach et al. 2012 for evidence suggesting that toddlers’ helping is motivated by other‐oriented and prosocial concerns).

These findings suggest sharing is rewarding early in development, and support claims that the “warm glow” from giving and other prosocial acts is a proximate mechanism for prosociality (Aknin et al. 2018). All that said, several of the key papers in this literature relied on very small samples that were not adequately powered to precisely estimate the effects of giving on happiness. For instance, Aknin, Hamlin, et al. (2012) tested 20 toddlers and reported effect sizes of *d* = 1.35 for costly giving versus receiving, *d* = 0.88 for non‐costly giving versus receiving, and *d* = 0.46 for costly versus non‐costly giving. Similarly, Aknin et al. (2015) tested 20 children and observed the same pattern, with *d* = 0.83, 0.46, and 0.30 for these respective contrasts. Although these studies provide important initial evidence that giving is emotionally rewarding early in development, their small sample sizes limit the precision with which effect sizes can be estimated, leaving open questions about the robustness and replicability of these effects. Furthermore, in nearly every study to date there has been a clear alternative explanation as to why children seemed happier to give than to receive: the social value of following the directions of an adult. That is, in previous studies children performing giving actions were always directly instructed to do so by a friendly experimenter, someone the children (presumably) wished to please. Given that young children are exquisitely attentive to their societies’ norms and what others wish them to do (Botto and Rochat 2018, 2019; Engelmann et al. 2012), children's positive responses after giving could have stemmed from an inherent pleasure derived from following the “norms” of the game (which, coincidentally, involved giving). If children in past studies were happy not due to the true prosociality of their acts but because they were performing a prompted, socially‐valued action, then this would suggest past work demonstrating the “warm glow” of giving in very young children may not reflect some more human‐general motivation for prosociality (see also Dahl and Brownell 2019 vs. Warneken and Tomasello 2015 for related arguments).

**FIGURE 1:**
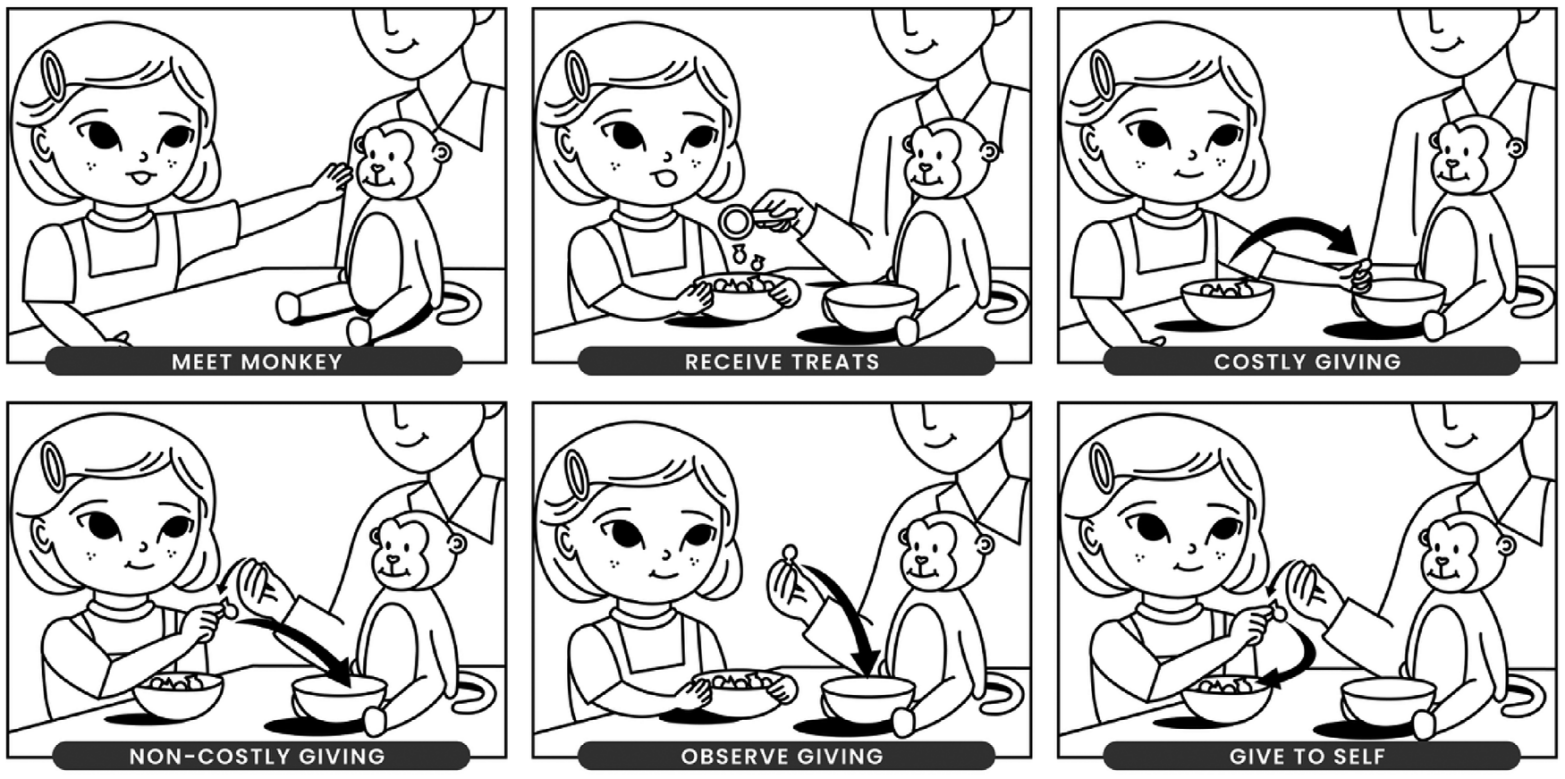
Overview of study procedure. Toddlers were first introduced to a puppet (“meet Monkey”) and then received eight treats (“receive treats”). They then participated in four counterbalanced phases: costly giving (giving one of their own treats to Monkey), non‐costly giving (giving a treat provided by the experimenter to Monkey), observe giving (watching the experimenter give a treat to Monkey), and give to self (giving a treat provided by the experimenter to themselves). In all phases, Monkey responded identically upon receiving a treat.

The main purpose of this study was to examine whether toddlers exhibit the warm glow from giving in a high‐powered study that addresses this potential alternative explanation. We replicated and extended the original design that first demonstrated the warm glow effect (Aknin, Hamlin, et al. 2012), by adding a phase in which toddlers were instructed to give a treat *to themselves* (Figure 1). If the warm glow experienced by children in past work was based on their desire to follow instructions and not on the prosocial nature of giving, then toddlers should find *any* prompted activity rewarding, including any prompted act of giving, whether it is prosocial (directed to others) or not (directed to themselves). However, if the warm glow effects are instead due to the rewarding nature of prosocial acts, then toddlers should only express more happiness after giving to someone else. Importantly, we recruited a large, well‐powered sample (*N* = 134) across a relatively wide age range (16.57–23.77 months), including children who have only recently begun sharing food, to provide a robust analysis of toddlers' sharing behaviors.

Based on prior research (Aknin, Hamlin, et al. 2012, Aknin et al. 2015; Fast et al. 2023; Song et al. 2020), we predicted toddlers would exhibit greater happiness after giving than after receiving or observing others give. Similarly, based on past work (Aknin, Hamlin, et al. 2012; Aknin et al. 2015; Song et al. 2020), we predicted toddlers would exhibit greater happiness after costly giving (e.g., giving treats from their own supply) versus non‐costly giving (e.g., giving treats an experimenter supplied). Finally, to test our novel hypothesis that the warm glow of giving is uniquely driven by prosocial behavior and not simply following instructions, we predicted toddlers would show greater happiness after giving to others versus themselves.

## 2 Method

### 2.1 Participants

Participants were 134 full‐term and healthy toddlers (66 female; *M*_age_ = 20.50 months, age range = 16.57–23.77 months) from middle‐class families living in a North American city. Children were recruited from a database based on hospital birth records. Most participants were from Caucasian and Asian families, representative of the population in the area; see Supplementary Materials (SM) 1 for details on child ethnicity and caregiver education. An a priori power analysis revealed that this sample size provided a power (1 − *β*) above 0.99 to detect a medium effect (*η_p_* ^2^ ​ = 0.06) in happiness ratings between different phases of the task. Twenty additional children were excluded for failure to complete the warm‐up (*N* = 16), experimenter errors (*N* = 2), fussiness (*N* = 1), and parental interference (*N* = 1). The study was conducted in accordance with the ethical standards of the American Psychological Association. The procedure was approved by the university's behavioral research ethics board. Clinical study registration was not required for this study. Written informed consent was obtained from a caregiver or guardian for each child before any data collection.

### 2.2 Procedure

Children sat on their caregiver's lap across the table from the experimenter throughout the study. Caregivers wore headphones and listened to music, and were asked to keep their eyes closed during the testing phase and avoid interacting with the child. These measures were implemented to minimize the possibility of caregivers influencing the child's behavior, ensuring that the child's responses were independent and not guided towards cooperation or obedience.

#### 2.2.1 Warm‐Up

The warm‐up phase was designed to familiarize children with the testing setup, to introduce them to puppets, and to demonstrate that puppets liked eating treats. The procedure was the same as in Aknin, Hamlin, et al. (2012). Children were presented with three stuffed animals (lion, panda, giraffe) and were told that the puppets liked eating treats. The experimenter then gave each puppet and the child an empty bowl. The puppets’ bowls had a false bottom, allowing them to “eat” their treats. Next, the experimenter gave each puppet and the child a treat (either goldfish or teddy graham crackers; treat type counterbalanced across the warm‐up and testing phases). The puppets ate the treats by pushing them into the bottom of the bowl with their noses while saying, “Oh, yum, yum, yum.” The experimenter then placed a “common” bowl with additional treats next to the child's bowl and encouraged the child to give one treat to each puppet and one treat to themselves. The puppets “ate” the treats given by the child in the same manner as they did when receiving treats from the experimenter.

#### 2.2.2 Testing

After the warm‐up phase, children participated in a six‐part treat giving game adapted from the paradigm used in Aknin, Hamlin, et al. (2012). In the “meet Monkey” phase, the experimenter introduced a new puppet (“Monkey”) to children and asked them to pet its nose. This phase allowed children to interact with the puppet. Monkey was then given a bowl. The experimenter told the child that Monkey liked treats and explained that neither the child nor Monkey had any treats. In the “receive treats” phase, the experimenter appeared to find a bowl containing twelve treats (goldfish or teddy graham crackers) and poured eight treats into the child's bowl, providing the child with valuable resources. The child then participated in four counterbalanced phases. In the “costly giving” phase, the child was asked to give one of their own treats to Monkey. Here, giving to Monkey incurred a personal cost. In the “non‐costly giving” phase, the experimenter “found” another treat (hidden in an opaque bowl underneath the table) and asked the child to give it to Monkey. Here, giving to Monkey did not involve a personal cost because the treat was provided by the experimenter. In the “observe giving” phase, the experimenter “found” another treat and gave it to Monkey. This condition allowed children to witness Monkey receiving a treat, without resource sacrifice or direct interaction with Monkey. Finally, in the “give to self” phase, the experimenter “found” another treat and asked the child to give this treat to themselves. This condition allowed children to actively behave following an instruction from an experimenter they might wish to please, without direct interaction with Monkey. Monkey responded in the same way each time it received a treat (saying “Oh, yum, yum, yum” while pushing the treat into the bottom of the bowl with its nose). If the child failed to engage in any of the requested actions, the experimenter asked again and provided prompts.

### 2.3 Happiness Coding

#### 2.3.1 Child Happiness

Children's facial responses in each phase were coded from video recordings of the study. Three independent coders rated the children's emotional reactions to each action on a seven‐point Likert scale. The scale was defined as follows: 1 = *not at all happy*, 2 = *big frown*, 3 = *small frown*, 4 = *neutral*, 5 = *small smile*, 6 = *big smile*, and 7 = *very happy (laughing)*; see SM 2 for coding instructions and example frames. Importantly, happiness was operationalized based on observable facial responses and was intended to capture a general positive affective state, which may reflect a blend of positive emotions rather than a single discrete emotion. The ratings from the coders for each phase were averaged (average *α* = 0.80). Coders were kept blind to the study hypotheses. To ensure that happiness ratings were not influenced by phase order and contextual cues, coders viewed shuffled video clips featuring only the child's face.

#### 2.3.2 Puppet Happiness

To explore whether children's happiness in the current study was related to the recipient's reaction, we coded the puppet's happiness during the task. Coders viewed video clips of Monkey from each of the six phases described above, and rated puppet happiness in each phase on a seven‐point Likert scale ranging from 1 (*not at all happy*) to 7 (*very happy*). To ensure that coders were blind to the participant's identity, black rectangles were added to the video clips to obscure the child (see SM 3 for coding instructions and interrater reliability).

### 2.4 Analysis Plan

To examine whether children's displays of happiness differed between phases, we performed a linear mixed‐effects model analysis using R (version 4.4.0; R Core Team 2024) and the *lme4* package (Bates et al. 2015). Happiness ratings were entered as the dependent variable. Task phase, phase order, age, sex, and the interactions between phase and age as well as between phase and sex were entered as fixed effects. Following the analytical approach used by Aknin, Hamlin, et al. (2012), and to enable comparisons between key conditions and baseline phases (e.g., receiving treats), all six phases were included in the primary model. A supplementary analysis focusing on the four counterbalanced phases is reported in SM 4. Participant was included as a random effect, with both a random intercept and a random slope for phase order. Omnibus tests of fixed effects were performed using Kenward‐Roger‐adjusted F‐tests via the *lmerTest* package (Kuznetsova et al. 2017). For significant effects, we evaluated robustness with likelihood ratio tests, comparing a full model with a nested model excluding the effect of interest. Planned pairwise comparisons between phases were conducted using model‐based contrasts estimated with the *emmeans* package (Lenth and Piaskowski 2025), with degrees of freedom computed using Kenward–Roger approximations. Model assumptions (including linearity, normality of residuals, and homoscedasticity) were confirmed through visual inspection of residual and Q‐Q plots.

To examine whether children's positive responses in different phases were attributable to the puppet's reaction to receiving treats, we performed an additional analysis on phases where Monkey received treats (costly giving, non‐costly giving, observe giving). First, a linear mixed‐effects model analysis was performed to examine whether differences between these three phases still held when puppet happiness was controlled for. Second, we examined whether children's happiness was correlated with puppet happiness within each phase.

All study data and analysis script are publicly available on the Open Science Framework (OSF) at https://osf.io/bn5fk/?view_only=7106040d2b674fc28e0c3c758c6f4215.

## 3 Results

### 3.1 Prosocial Giving Led to Greater Happiness in Toddlers

**FIGURE 2:**
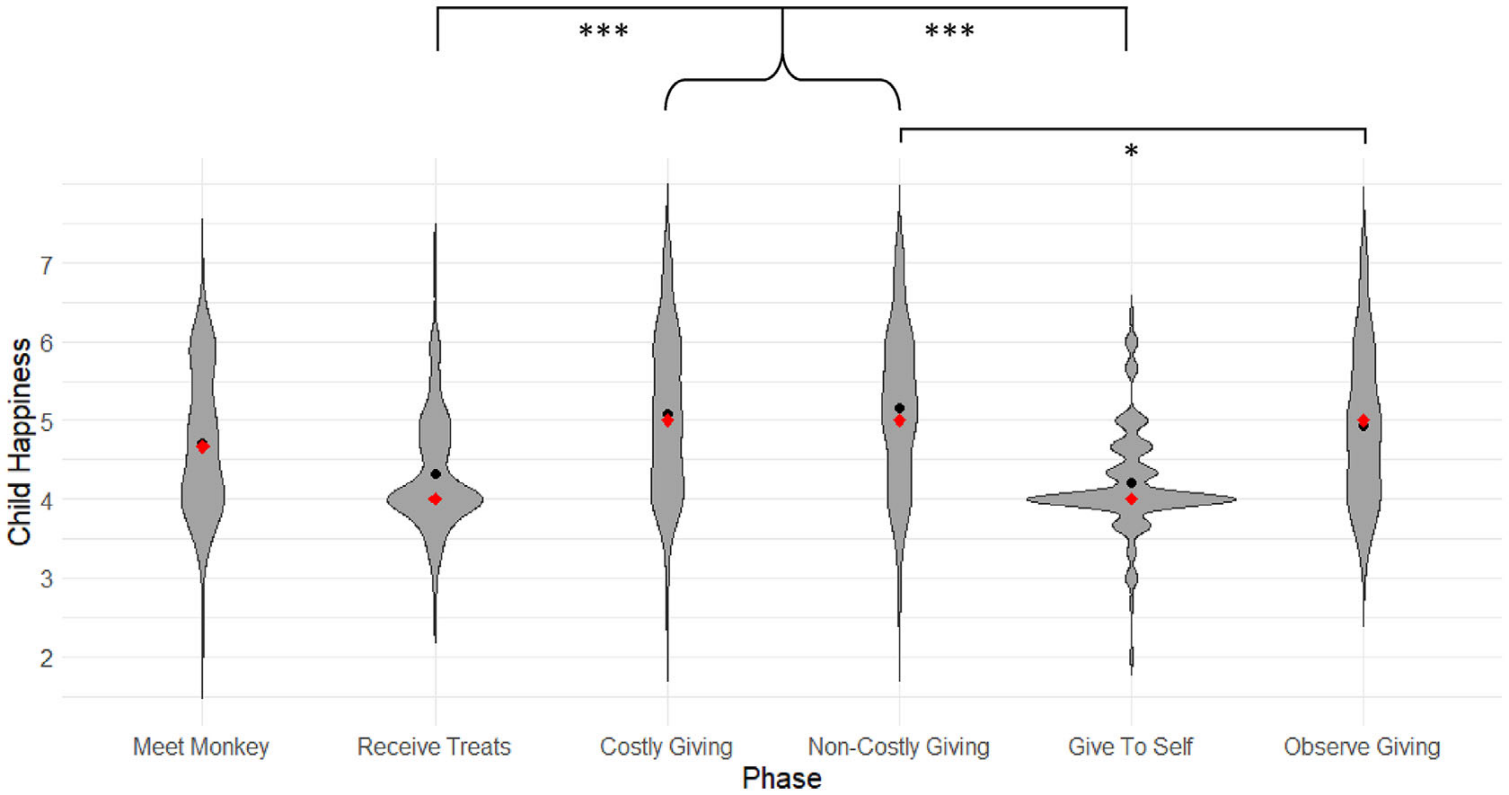
Happiness ratings during different phases of the treat giving game. Black dots indicate average scores and red dots indicate median scores. *p < 0.05, ***p < 0.001.

A linear mixed‐effects analysis of child happiness revealed a significant main effect of phase, *F*(5, 563.32) = 38.33, *p* < 0.001 (see SM 5 for a likelihood ratio test confirming the robustness of this effect). No other omnibus fixed effects were significant, including age (see SM 6 for details), sex, phase order, or the interactions between phase and age and between phase and sex, *p*s ≥ 0.218. Planned comparisons between phases revealed that, consistent with our first hypothesis, toddlers exhibited greater happiness after giving their own treats (costly giving, *M* = 5.07, *SD* = 0.98, *SE* = 0.09) or the experimenter's treats (non‐costly giving, *M* = 5.16, *SD* = 0.98, *SE* = 0.08) to a puppet than after receiving treats themselves (receive treats, *M* = 4.32, *SD* = 0.71, *SE* = 0.06; costly giving versus receive treats: *t*(539) = 6.26, *p* < 0.001, *d* = 0.98, 95% CI [0.67, 1.28]; non‐costly giving versus receive treats: *t*(540) = 6.96, *p* < 0.001, *d* = 1.10, 95% CI [0.78, 1.41]; see Figure 2). These findings replicate a large body of past work suggesting that toddlers find giving more rewarding than receiving. Additionally, consistent with prior findings with preschoolers (Fast et al. 2023), toddlers displayed more happiness after sharing resources provided by the experimenter (non‐costly giving) than after observing the experimenter give treats to the puppet (observe giving, *M* = 4.94, *SD* = 0.95, *SE* = 0.08; non‐costly giving versus observe giving: *t*(584) = 2.41, *p* = 0.016, *d* = 0.30, 95% CI [0.05, 0.54]), suggesting that actively engaging in non‐costly sharing is more rewarding for toddlers than merely observing a recipient of sharing emote positively. Somewhat surprisingly, this effect did not extend to costly giving, costly giving versus observe giving: *t*(591) = 1.43, *p* = 0.154, *d* = 0.18, 95% CI [−0.07, 0.42]. Contrary to our second hypothesis, we did not detect differences in toddlers’ happiness after engaging in costly versus non‐costly acts of giving, *t*(596) = −0.97, *p* = 0.331, *d* = −0.12, 95% CI [−0.37, 0.12], suggesting that the previously observed happiness boost related to personal sacrifice may not be robust. When we averaged toddlers’ happiness after costly and non‐costly giving into a composite measure, it was significantly higher than their happiness after observing sharing, *t*(443) = 2.05, *p* = 0.041, *d* = 0.25, 95% CI [0.01, 0.50], highlighting the emotional benefits of actively engaging in prosocial behavior versus merely observing it.

That toddlers did not show a distinction between costly and non‐costly giving in this sample renders the alternative explanation raised above, that toddlers are simply happy to follow an experimenter's instructions, particularly plausible. However, toddlers who followed instructions from the experimenter and gave to themselves were not rated as particularly happy (give to self, *M* = 4.21, *SD* = 0.65, *SE* = 0.06); indeed, toddlers were no happier after receiving stemming from following instructions (giving to self) than they were after receiving in a windfall (receive treats), *t*(538) = −1.78, *p* = 0.076, *d* = −0.28, 95% CI [−0.58, 0.03]. In contrast, children were consistently happier after giving to others versus giving to themselves (costly giving versus give to self: *t*(588) = 10.09, *p* < 0.001, *d* = 1.25, 95% CI [1.00, 1.50]; non‐costly giving versus give to self: *t*(589) = 11.06, *p* < 0.001, *d* = 1.37, 95% CI [1.12, 1.63]). Together, these results strongly suggest that giving only leads to greater happiness in toddlers when it is directed toward others, effectively ruling out the “following instructions” alternative explanation for past work.

### 3.2 Toddlers’ Happiness Was Unrelated to Recipient's Enthusiasm

To examine whether toddlers’ positive responses in the current study were influenced by the recipient's reaction to receiving treats, we coded the puppet's happiness during phases where they received treats (i.e., costly giving, non‐costly giving, and observe giving) and performed an additional analysis on these phases. Linear mixed‐effects analysis found a significant main effect of phase, *F*(2, 260.98) = 6.11, *p* = 0.003. No other omnibus fixed effects were significant, *p*s ≥ 0.077. Importantly, puppet happiness did not predict child happiness across phases, *F*(1, 338.20) = 0.67, *p* = 0.414. After controlling for puppet happiness, toddlers were rated as happier after actively giving than after merely observing giving, costly giving versus observe giving, *t*(262) = 2.24, *p* = 0.026, *d* = 0.28, 95% CI [0.03, 0.53]; non‐costly giving versus observe giving, *t*(262) = 3.45, *p* < 0.001, *d* = 0.44, 95% CI [0.19, 0.70]; in fact, controlling for puppet happiness increased observed effect sizes. Within each phase, puppet happiness was not correlated with child happiness, |*r*s| ≤ .11, *p*s ≥ 0.203. These findings suggest that variations in toddlers’ happiness in the current study were unlikely to be attributable to differences in puppet enthusiasm and more likely reflect the warm glow of giving to others.

## 4 Discussion

Across cultures, people willingly share valuable resources with others (Helliwell et al. 2025). One explanation for the prevalence of sharing behaviors is that they are intrinsically rewarding, reinforcing subsequent prosocial acts. To understand the roots of human prosociality, it is essential to clarify whether there are causal links between prosocial acts and positive emotions early in ontogeny. Here, we provide evidence that, shortly after toddlers begin to share, they find giving to others more rewarding than receiving resources, either when provided by others or themselves. Additionally, sharing resources provided by the experimenter elicited greater happiness than did passively observing the experimenter share. Interestingly, toddlers’ happiness did not differ between situations where they gave their own treats (costly giving) versus treats provided by the experimenter (non‐costly giving), suggesting the happiness boost previously associated with personal sacrifice (e.g., Aknin, Hamlin, et al. 2012; Aknin et al. 2015) may not be a consistent effect, and both costly and non‐costly sharing may be similarly rewarding. Finally, the emotional benefits of giving were unrelated to puppet enthusiasm in the current study, suggesting emotional reward comes from performing kind actions rather than contagion of positive emotions. Together, our findings support the hypothesis that generous sharing is emotionally rewarding early in development.

Toddlers in the current study showed greater happiness after giving than receiving resources, replicating the original findings (Aknin, Hamlin, et al. 2012, Aknin et al. 2015) with a sample size more than six times larger. Importantly, while toddlers’ sharing in these studies was prompted by the experimenter, our results demonstrate (prompted) giving to others produced greater happiness than (prompted) giving to oneself, suggesting the emotional benefits of sharing are driven by its prosocial nature, rather than by more general aspects of social interactions or following instructions. Together with the results from previous studies with toddlers (Aknin, Hamlin, et al. 2012, Aknin et al. 2015; Song et al. 2020) and older children (Fast et al. 2023; Song et al. 2020), our findings further support the hypothesis that emotional rewards may serve as a crucial mechanism for promoting prosociality across development.

While prosocial behaviors are likely shaped by experience and socialization processes, the early emergence of these emotional benefits from giving, and the selectivity of these benefits to prosociality versus socialization‐relevant features of the act (e.g., following instructions) suggests sharing may be intrinsically rewarding when it first appears. Notably, despite the relatively wide age range of our sample (16.57–23.77 months), age did not moderate the observed differences in child happiness between phases (see SM 6). This suggests the emotional rewards of sharing do not increase with age, consistent with the hypothesis that sharing's emotional benefits emerge early in life and are not solely the result of socialization.

Past research suggests that actively performing prosocial actions leads to greater happiness (“warm glow”) than observing others act prosocially (Andreoni 1989). Here, toddlers displayed greater happiness after sharing resources provided by the experimenter than after observing the experimenter share, replicating similar findings in preschoolers (Fast et al. 2023). One explanation for this effect is that being recognized for performing prosocial actions improves one's reputation, leading to greater satisfaction. However, research has demonstrated toddlers are equally likely to help others regardless of whether they are observed (Hepach et al. 2017; Warneken and Tomasello 2013), and that reputational concerns do not begin to impact prosocial behaviors until later in preschool (for a review, see Engelmann and Rapp 2018). This aligns with a previous study on toddlers’ giving in which happiness after giving did not vary based on whether toddlers were praised (Song et al. 2020). Similarly, adults experience emotional rewards from giving even when their kind acts are unknown to others (Aknin, Barrington‐Leigh, et al. 2013, Aknin et al. 2020). Together with our findings that performing an action directed by an adult, even when that action involves giving, is only rewarding if the action is prosocial (e.g., giving to someone else), this work strongly suggests reputational concerns are not a primary motivation for toddlers’ prosocial behaviors (see also Hepach et al. 2022). Thus, toddlers’ happiness may reflect an intrinsic reward for performing prosocial actions.

Although active giving generated greater happiness than observing giving, it is worth noting that observing giving still led to greater happiness than receiving, *t*(539) = 5.08, *p* < 0.001, *d* = 0.80, 95% CI [0.49, 1.11], and giving to self, *t*(593) = 8.65, *p* < 0.001, *d* = 1.08, 95% CI [0.83, 1.32]. This suggests toddlers derive satisfaction from seeing others’ needs met, regardless of who fulfills the needs. Past work on instrumental helping has shown that children's pupil size (indexing sympathetic arousal) increased when observing someone in need of help, and this increase predicted subsequent helping (Hepach et al. 2019). Crucially, children's pupil size decreased once help was provided, whether they helped or a third party did. These results suggest children are primarily motivated to see others’ needs met (see Hepach et al. 2017, for a review). Similarly, in the context of resource distribution, Fast and colleagues (Fast et al. 2023) found 5‐year‐old children showed greater happiness from sharing when they could see the beneficiary's positive responses, suggesting beneficiary satisfaction contributes to children's happiness (as, indeed, to adults’; Aknin, Dunn, et al. 2013; Morelli et al. 2015). Notably, because in the current study toddlers' happiness was unrelated to puppet enthusiasm and held when controlling for it, it is unlikely that toddlers were merely experiencing emotional contagion. Nonetheless, seeing others receive or have their needs met appeared to increase toddlers' happiness relative to baseline/receiving themselves.

Our findings should be interpreted in light of limitations. First, our sample was not cross‐cultural (but see Aknin et al. 2015; Song et al. 2020) and our analyses relied on observational coding of facial expressions. Future research should recruit more diverse samples and incorporate additional measures of emotion (e.g., pupil dilation, electrodermal activity, motion‐capture‐based measures of posture elevation) to enhance generalizability. Second, although costly giving did not elicit greater happiness than non‐costly giving in the present sample of toddlers, it remains possible that sensitivity to the personal cost of giving emerges later in development, as children's understanding of resource value strengthens. Future work incorporating a broader age range, larger samples, and more direct manipulations of perceived cost or value could test whether the emotional rewards of giving become increasingly contingent on personal sacrifice across development. Third, it is important to note that the experimenter was instructed to keep the puppet's happiness as constant as possible across phases in which the puppet received treats and was quite successful in doing so. Indeed, coder ratings showed limited variability in puppet's happiness with 92.54% of puppet happiness scores falling between 5 and 6. Thus, although the absence of a correlation between child happiness and puppet happiness suggests that differences in child happiness across phases in the present study cannot be explained by puppet happiness, this does not rule out the possibility that puppet happiness *can* influence children's emotional responses. In fact, experiments conducted with adults show that people tend to experience greater emotional rewards from giving when they can see or understand how their actions have benefited others (e.g., Aknin, Dunn, et al. 2013). Future research should test this possibility in young children by systematically varying puppet happiness to examine its effects on child happiness. Despite these limitations, this study offers strong evidence that, soon after sharing behaviors emerge, young children experience greater reward from giving to others than from receiving, observing giving, and giving to themselves. These findings support the hypothesis that prosocial behaviors are intrinsically rewarding from soon after they emerge, creating a self‐reinforcing basis for widespread human cooperation.

## Author Contributions

**Enda Tan**: writing – original draft preparation, writing – review and editing, conceptualization, formal analysis, investigation, software, visualization. **Julia Van de Vondervoort**: writing – review and editing, conceptualization, methodology, investigation. **Jeneesha Dhaliwal**: writing – review and editing, formal analysis, software, validation, data curation. **Lara B. Aknin**: writing – review and editing, conceptualization, methodology, resources, supervision, funding acquisition. **J. Kiley Hamlin**: writing – review and editing, conceptualization, methodology, resources, supervision, funding acquisition.

## Funding

This work was supported by the Social Sciences and Humanities Research Council of Canada (Grant GR002225) to J. Kiley Hamlin.

## Ethics Statement

The study was approved by the Research Ethics Boards at the University of British Columbia and conducted in accordance with the ethical standards of APA and the provisions of the World Medical Association Declaration of Helsinki. Informed consent was obtained from the parents or legal guardians of all participants.

## Conflicts of Interest

The authors declare no conflicts of interest.

## Permission to Reproduce Material From Other Sources

No material from other sources has been reproduced or reused in this manuscript.

## Supporting information




**Supporting File 1**: desc70171‐sup‐0001‐SupMat.docx

## Data Availability

All primary data are publicly available at the Open Science Framework (OSF; https://osf.io/bn5fk/?view_only=7106040d2b674fc28e0c3c758c6f4215). All analysis scripts are also publicly accessible at the same OSF repository.
