# Supplementary material for: Toddlers Are Happier Giving to Others Than to Themselves
Source: Dev Sci. 2026 Mar 17;29(3):e70171. doi: 10.1111/desc.70171 (PMC12994117; doi:10.1111/desc.70171)
Supplement: Supplementary file 1 — Supporting File 1: desc70171‐sup‐0001‐SupMat.docx [file DESC-29-e70171-s001.docx]

**Supplementary Materials**

**1. Participant demographics**

Table S1. Breakdown of child ethnicity

| **Child Ethnicity** | **N** | **%** |
| --- | --- | --- |
| East Asian / Southeast Asian | 11 | 8.21 |
| White | 88 | 65.67 |
| Mixed race | 35 | 26.12 |

Table S2. Breakdown of caregiver education level

| **Caregiver education level** | **N** | **%** |
| --- | --- | --- |
| Some high school | 1 | 0.75 |
| High school | 1 | 0.75 |
| Some college/university | 4 | 2.99 |
| College certificate/diploma | 11 | 8.21 |
| Trade school diploma | 5 | 3.73 |
| Bachelor’s degree | 44 | 32.84 |
| Master’s degree | 33 | 24.63 |
| Doctoral degree | 7 | 5.22 |
| Professional degree | 26 | 19.40 |
| Missing values | 2 | 1.49 |

**2. Child happiness coding instructions and example video frames**

Coding Instructions

All coders had prior experience interacting with toddlers (e.g., through frequent babysitting or regular interaction with young family members). Before coding began, coders viewed all video clips for each child to gain an overall sense of that child’s expressivity. This step was necessary because toddlers vary widely in how strongly they display emotion. For less reactive children, expressions such as a small smile may be very brief or subtle (e.g., a slight upturn of the lips) and sometimes require multiple viewings to detect.

For each phase, coders assigned a single happiness rating using a 7-point scale, ranging from 1 (*not at all happy*) to 7 (*very happy*). Half-point ratings were not permitted, and even the smallest detectable smile was coded as a 5. A phase was coded as neutral (4) only when the child’s face remained entirely flat and showed no affect for the duration of the phase. Any detectable positive expression, even if brief or subtle, was coded as a 5. Coders recorded the most extreme emotional response observed within each phase; for example, if a child was mostly neutral but briefly laughed, the phase was coded as a 7.

Coders were permitted to view each clip as many times as needed to make an accurate judgment. Ratings for each phase were entered into a standardized Excel coding sheet. If a phase or video was missing, the corresponding cell was left blank. When coders were uncertain about a rating, they coded the child’s happiness to the best of their ability and documented their reasoning in the comments column. Any major questions or issues that arose during coding were communicated to the full coding team to ensure consistency across coders.

Cropping Instructions

To ensure that coders remain blind to the phase being coded, videos were cropped or masked (e.g., using black bars) to display only the child’s face and the top of the shoulders, while excluding the child’s hands and any objects on the table (e.g., bowls or puppets). Each video was checked to confirm that the black bars or cropping did not obscure any part of the child’s face at any point during the phase. Prior to being provided to coders, videos were renamed so that phase identity could not be inferred from the file names. In addition, phases were presented in a random order; coders were not shown phases in their original temporal order and were not systematically exposed to any particular phase first (e.g., Costly Giving).

Example Video Frames


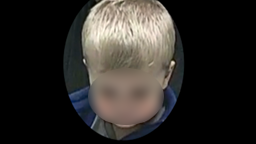

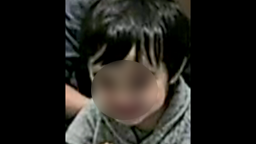


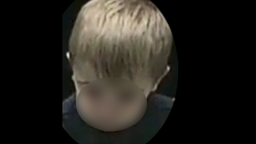


*Note*. Facial features have been blurred to remove identifiable information.

**3. Puppet happiness coding instructions and interrater reliability**

Coding Instructions

Coding procedures for puppet happiness closely paralleled those used for child happiness. Puppet/experimenter videos were masked (e.g., with black rectangles) to obscure the child participant’s identity and were shuffled prior to coding. For each phase, coders assigned a single puppet happiness rating using a 7-point scale ranging from 1 (*not at all happy*) to 7 (*very happy*). Half-point ratings were not permitted. Coders recorded the most extreme emotional expression displayed by the puppet during each phase.

Coders were allowed to view each clip as many times as needed to make an accurate judgment. Ratings for each phase were entered into a standardized Excel coding sheet. If a phase or video was missing, the corresponding cell was left blank. When coders were uncertain about a rating, they coded the puppet’s happiness to the best of their ability and documented their reasoning in the comments column. Any major questions or issues that arose during coding were communicated to the full coding team to ensure consistency across coders.

Interrater reliability and agreement

To assess the reliability of puppet happiness ratings during the costly giving, non-costly giving, and observe giving phases, a second coder independently rated 52.99% of the sample (N=71). Interrater reliability was low to modest across indices: Krippendorff’s alpha was low (α=.22), and a two-way random-effects intraclass correlation coefficient (absolute agreement; single measures) was modest, ICC(agreement, 1) = .24, 95% CI [.11, .36]. Quadratically weighted Cohen’s κ was similarly modest (κ=.24).

These coefficients likely underestimate the practical consistency of the coders because the happiness ratings showed substantial range restriction. Restricted and skewed score distributions can attenuate variance-based reliability statistics (e.g., Krippendorff’s α, ICC) and can also yield low κ values even when raters frequently agree (Erosheva et al., 2021; Fife et al., 2012; Sackett et al., 2002). This restriction was expected given that these ratings were drawn from phases in which Monkey received treats and the experimenter was instructed to remain consistently positive. Indeed, across all 426 ratings (71 participants × 3 phases × 2 coders), scores fell only between 4 and 7, with 92.96% of ratings at 5 or 6 (see Table S3).

Table S3. Distribution of puppet happiness ratings across the two raters and three phases

| **Score** | **N** |
| --- | --- |
| 1 | 0 |
| 2 | 0 |
| 3 | 0 |
| 4 | 11 |
| 5 | 209 |
| 6 | 187 |
| 7 | 19 |

Despite the low-to-modest reliability coefficients, coders demonstrated high absolute agreement. Across the 213 paired ratings, coders assigned identical scores in 48.83% of cases (104/213) and differed by no more than one point in 96.71% of cases (206/213; see Table S4). Thus, although conventional reliability indices were attenuated (likely due to restricted range) coders’ ratings were highly consistent in practical terms, with discrepancies almost always limited to a single scale point.

Table S4. Distribution of interrater differences in puppet happiness ratings across phases

| **Difference score**  **(Rater 1 − Rater 2)** | **N** |
| --- | --- |
| -1 | 37 |
| 0 | 104 |
| 1 | 65 |
| 2 | 7 |

**4. Linear mixed-effects analysis of child happiness across the four counterbalanced phases**

Replicating the primary analysis, a linear mixed-effects analysis of child happiness across the four counterbalanced phases (costly giving, non-costly giving, give to self, observe giving) found a significant main effect of phase, *F*(3, 387.46)=55.38, *p*<.001. No other fixed effects were significant, including age, sex, phase order, or the interactions between phase and age and between phase and sex, *p*s≥.223. Planned comparisons further replicated the primary analysis. Toddlers showed greater happiness after sharing resources provided by the experimenter (non-costly giving) than after observing the experimenter give treats to the puppet, *t*(382)=2.83, *p*=.005, *d*=0.36, 95% CI [0.11, 0.61]. This effect did not extend to costly giving; happiness following costly giving did not differ significantly from observing giving, *t*(389)=1.72, *p=*.087, *d*=0.22, 95% CI [-0.03, 0.47]. There was also no difference in toddlers’ happiness following costly versus non-costly giving, *t*(391)=-1.10, *p*=.273, *d*=-0.14, 95% CI [-0.39, 0.11]. Children were consistently happier after giving to others than after giving to themselves, both for costly giving, *t*(387)=10.47, *p*<.001, *d*=1.33, 95% CI [1.07, 1.60], and for non-costly giving, *t*(388)=11.56, *p*<.001, *d*=1.47, 95% CI [1.21, 1.74]). Observing giving led to greater happiness than giving to self, *t*(390)=8.72, *p*<.001, *d*=1.11, 95% CI [0.85, 1.37]. Taken together, these results closely mirror the effects observed in the analysis including all six phases.

**5. Robustness check for the effect of phase**

As a robustness check of the omnibus effect of phase, we compared a full model and a reduced linear mixed-effects model using a likelihood ratio test. The full model included phase, phase order, age, and sex as fixed effects, with participant-specific random intercepts and random slopes for phase order. The reduced model was identical except that it omitted the fixed effect of phase. The full model provided a significantly better fit to the data than the reduced model, likelihood ratio test, *χ*²(5)=167.61, *p*<.001, confirming that the overall effect of phase was robust.

**6. The effects of age**

Omnibus tests indicated that age did not predict children’s happiness across phases, *F*(1, 133.16)=0.72, *p*=.397, nor did it moderate phase-related differences in happiness, *F*(5, 591.58)=1.25, *p*=.283 (see Figures S1-S2). Consistent with these results, estimated age slopes within each phase were small and all 95% confidence intervals included zero: meet monkey (*b*=-0.02, 95% CI [-0.10, 0.05]), receive treats (*b*=0.07, 95% CI [-0.01, 0.14]), costly giving (*b*=-0.00, 95% CI [-0.08, 0.08]), non-costly giving (*b*=0.02, 95% CI [-0.06, 0.10]), give to self (*b*=0.06, 95% CI [-0.02, 0.14]), and observe giving (*b*=0.01, 95% CI [-0.07, 0.09]). Age slopes did not differ across phases, *F*(5, 535.77)=1.25, *p*=.282, providing further support for the possibility that the relationship between age and child happiness did not differ across phases.

Figure S1. The relations between age and child happiness across all study phases


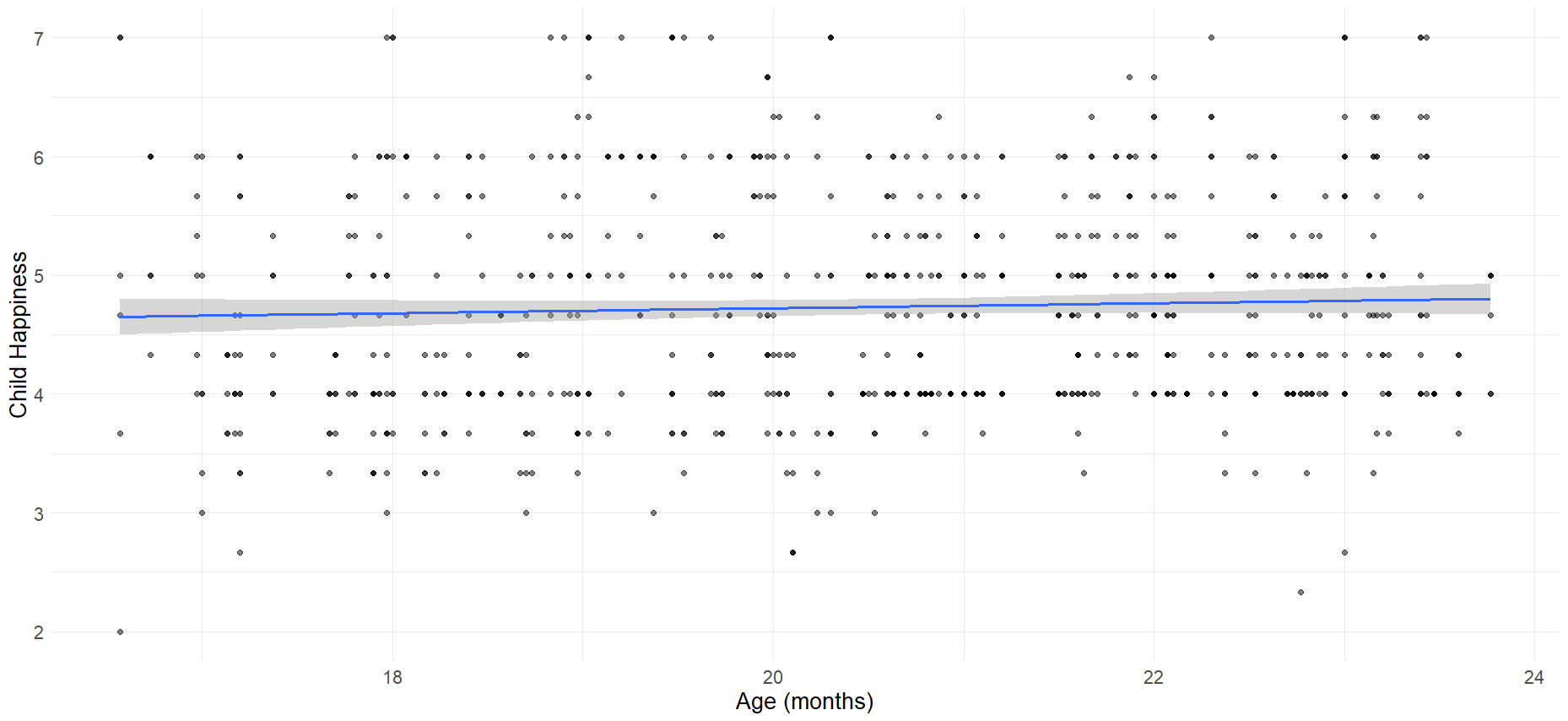


*Note*. This scatterplot pools happiness ratings across all phases. The blue line shows the overall linear trend in happiness with age. The grey ribbon shows the 95% confidence interval around that trend.

Figure S2. The relations between age and child happiness within each study phase


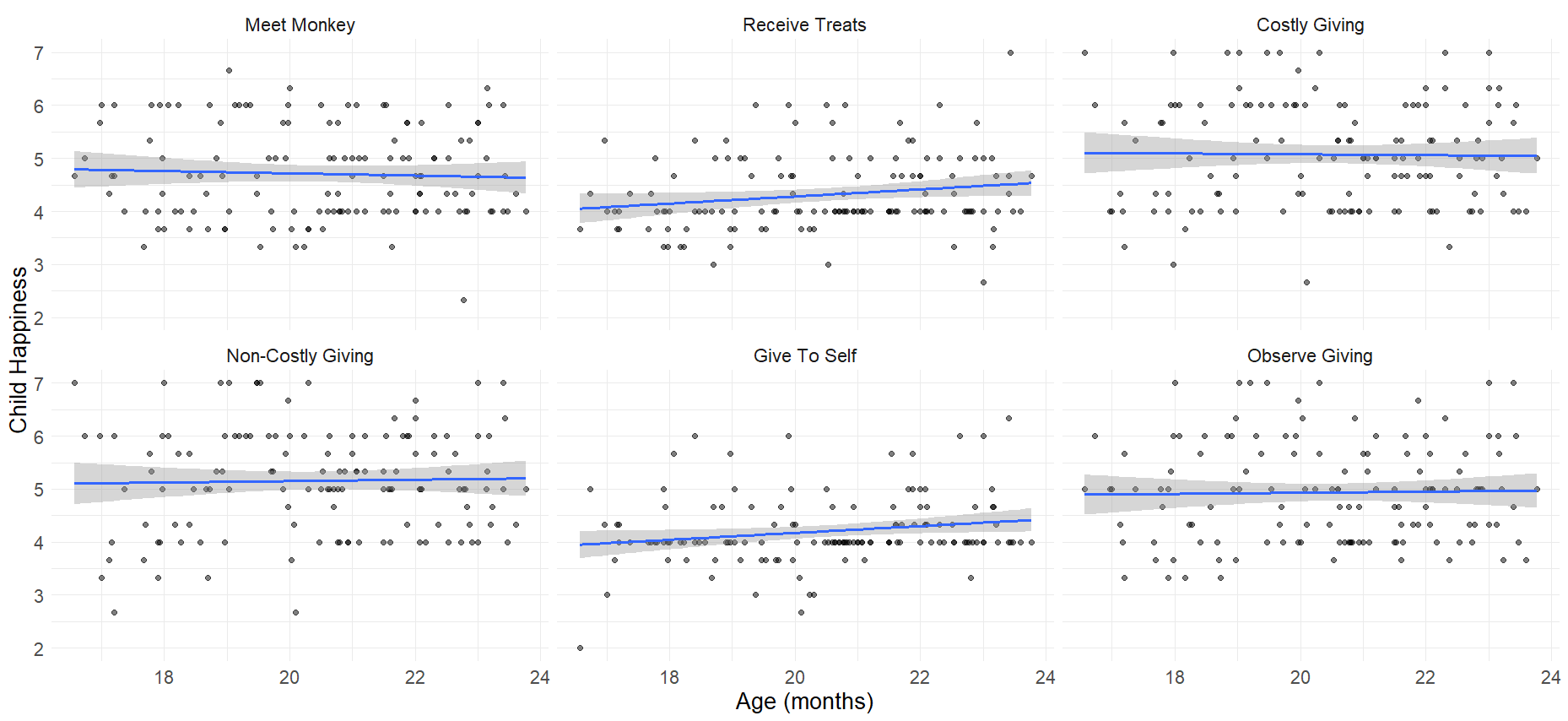


*Note*. These scatterplots show the age-happiness association separately for each phase, with a phase-specific fitted linear trend (blue line) and its 95% confidence interval (grey ribbon).
